# Investigations of remibrutinib in models pertinent to multiple sclerosis

**DOI:** 10.1016/j.neurot.2026.e00923

**Published:** 2026-05-14

**Authors:** Rajiv W. Jain, Atefeh Rayatpour, Kathleen Hagen, Marlene T. Morch, Gurleen Randhawa, Maryam Mobarakabadi, Xiangyu Zhang, Cenxiao Li, Maryam Nakhaei-Nejad, Fabrizio Giuliani, Bruno Cenni, Mengzhou Xue, V. Wee Yong

**Affiliations:** a Hotchkiss Brain Institute and the Department of Clinical Neurosciences, University of Calgary, Canada; b Division of Neurology, Department of Medicine, University of Alberta, Canada; c Novartis BioMedical Research, Basel, Switzerland; d Department of Cerebrovascular Diseases, The Second Affiliated Hospital of Zhengzhou University, Zhengzhou, China

**Keywords:** Multiple sclerosis, BTK inhibitor, T-bet+ memory B cells, Remibrutinib

## Abstract

Inhibitors of Bruton tyrosine kinase (BTK) are actively being pursued as potential disease-modifying therapies for multiple sclerosis (MS), as attested by several completed or ongoing Phase 3 clinical trials. Yet, key aspects of BTK inhibitors biology remain unclear. Here, we assessed the effects of remibrutinib, a BTK inhibitor in Phase 3 MS trials, in preclinical models of MS. Remibrutinib was evaluated in the myelin oligodendrocyte glycoprotein (MOG)35-55 model of experimental autoimmune encephalomyelitis (EAE) and in the lysolecithin model of demyelination. In culture, we assessed its ability to reduce tumor necrosis factor-α (TNF-α) elicited by immune complex-activation of primary human and mouse microglia, mouse macrophages, and human inducible pluripotent stem cell (iPS)-derived microglia. We also examined the impact of remibrutinib on T-bet^+^ memory B cells, increasingly implicated in neuroinflammation. Remibrutinib attenuated clinical disability of MOG-induced EAE, accompanied by decreased lesional burden in the spinal cord. In lysolecithin-induced demyelination, remibrutinib did not affect oligodendrogenesis in young mice with robust repair capacity, or in middle-aged mice where remyelination is deficient. Across microglia and macrophage cultures of human or murine origin, remibrutinib effectively reduced immune complex-induced TNF-α. Multiplex cytokine analyses showed that this reduction coincided with increased interleukin-10, suggesting a shift toward a more regulatory functional state. In T-bet^+^ memory B cells, remibrutinib decreased production of pro-inflammatory cytokines in culture and reduced expression of markers associated with T-bet^+^ memory B cells in mice. While Phase 3 clinical trial results in MS are awaited, the drug favorably affects inflammatory outcomes in MS models.

## Introduction

The pathophysiology of multiple sclerosis (MS) includes the infiltration of immune cells into the central nervous system (CNS) and the activation of CNS-resident microglia [1,2]. In active lesions, as well as in the rim of chronic active lesions, microglia and monocyte-derived macrophages constitute the majority of the immune cell repertoire [3,4]. Persistent pro-inflammatory and degeneration-associated microglia and macrophages are proposed to exacerbate progression of disability in MS by producing axonal injury and loss, demyelination, and failure of remyelination [2,4,5]. B cells also contribute to the pathology of MS lesions primarily through their capacity to activate T lymphocytes and myeloid cells, and secrete inflammatory and toxic factors from within the meningeal or perivascular barriers [6]. Thus, inhibiting the activity of B cells and macrophages and microglia is an attractive strategy to counter the progression of MS. Bruton's tyrosine kinase (BTK) is an intracellular enzyme and a key signaling node following B cell receptor and Fc receptor engagement in B cells and myeloid cells, respectively [7,8]. BTK activation regulates the proliferation, maturation and activity of B lymphocytes [9], but is not required for their survival [10]. Thus, BTK inhibitors should regulate activity of B cells without depleting their numbers as seen with the anti-CD20 monoclonal antibodies used in MS. Similarly, BTK mediates the pro-inflammatory activity of myeloid cells [11,12]. In healthy or normal-appearing white matter of human and murine CNS, BTK immunoreactivity is undetectable but its expression and activity are prominently elevated in lesions of MS and its models in hypercellular aggregates of microglia and macrophages [13]. Thus, pharmacological inhibitors of BTK may simultaneously normalize aberrant B cell and myeloid functions in MS without overtly depleting their numbers.

There has been immense interest in using small CNS-penetrant molecules such as BTK inhibitors in MS. To that end, there have been several completed or ongoing Phase 3 clinical trials in MS with five BTK inhibitors across the spectrum of MS [7,8,14]. Of the current available Phase 3 results, both evobrutinib (in EVOLUTION 1 and 2 trials) and tolebrutinib (in GEMINI 1 and 2) did not meet their primary outcome of reducing annualized relapse rate in people with relapsing MS relative to the active comparator teriflunomide [15,16]. Encouragingly, tolebrutinib lowered the progression of disability in non-relapsing SPMS compared to placebo in the HERCULES trial [17]. However, in a recent press release, tolebrutinib did not meet its primary endpoint in delaying time to onset of 6-month composite confirmed disability progression compared to placebo in primary progressive MS (https://www.sanofi.com/en/media-room/press-releases/2025/2025-12-15-06-05-00-3205094). For fenebrutinib, detailed results from Phase 3 trials in relapsing (Clinicaltrials.gov NCT04586023 and NCT04586010) and primary progressive MS (NCT04544449) are expected soon, with a press release by Roche on November 10, 2025, that the trials met their primary outcomes. The fourth BTK inhibitor, remibrutinib, has two ongoing Phase 3 trials in relapsing MS (NCT05156281 and NCT05147220) and an ongoing Phase 3 trial in secondary progressive MS (NCT07225504), and results are anticipated in 2026 and 2030, respectively. The fifth BTK inhibitor, orelabrutinib, is in a Phase 2 trial in relapsing MS (NCT04711148) and a Phase 3 trial in primary progressive MS (NCT07067463).

Despite the immense interest in BTK inhibitors clinically in MS, their preclinical studies in models of MS have lagged behind. While there is relatively more information for evobrutinib in preclinical models [[18], [19], [20]], there are isolated reports for tolebrutinib [21,22], fenebrutinib [23], and remibrutinib [24,25]. Available results of these BTK inhibitors should not be lumped together as a class activity, as there are differences to the inhibitors including their reversible or covalent irreversible nature of their antagonism for BTK, selectivity to BTK versus other enzymes, and pharmacokinetic properties such as the capacity to penetrate into the CNS necessary for microglia inhibition [7,21,25,26].

Here, we sought to assess the effects of remibrutinib in tissue culture and animal models relevant to MS. Remibrutinib has been assessed in human myelin oligodendrocyte glycoprotein (MOG) B and T cell-driven EAE, where it lowered myeloid cell and microglia driven neuroinflammation [24], but it has not yet been examined in B cell-independent MOG35-55 EAE or for potential reparative actions in the lysolecithin model of demyelination. Moreover, the activity of remibrutinib on primary cultures of murine or human macrophages and microglia, or on human inducible pluripotent stem cell-derived (iPSC) microglia is unreported. It also remains unknown as to the impact of remibrutinib in vivo and *in vitro* on T-bet^+^ memory B cells (also known as age-associated or atypical memory B cells) [27]. There is evidence of these cells accumulating within the CNS of people with MS [28,29] where they exhibit an abhorrent activation profile [30]. These cells are likely to be pathogenic in MS as T-bet expression in B cells is required for gamma herpes viruses to enhance EAE severity [31], and T-bet^+^ memory B cells have the capacity to recruit inflammatory T lymphocytes into the CNS [32]. This manuscript reports new knowledge of remibrutinib pertinent to MS.

## Materials and Methods

### Remibrutinib

Remibrutinib, also referred as LOU064, was provided by Novartis (Basel, Switzerland). For administration in mice, remibrutinib was constituted as a suspension for oral gavage in 0.5% methylcellulose and 0.5% Tween 80 in water. Previous work using human MOG EAE in mice, a B cell-dependent model [33], had shown good reduction of EAE scores with 30 mg/kg dose when remibrutinib was initiated at the time of MOG immunization [24]. In that study, BTK receptor occupancy in brain homogenates 1 h after the last dose of 30 mg/kg remibrutinib at day 28 of EAE was close to 100%. As well, the study showed that 1 h after 30 mg/kg in mice, the concentration of remibrutinib measured by liquid chromatography and mass spectrometry was 371 nM, 13.5 nM and 8.4 nM in blood, brain and cerebrospinal fluid, respectively [24].

For our study in MOG35-55 EAE, which does not involve significant activation of B cells [33], our initial experience using 30 mg/kg dose of remibrutinib did not consistently reduce EAE disability, so 60 mg/kg was used for subsequent EAE and lysolecithin experiments, with the exception of those in Fig. 5 (30 mg/kg). For remibrutinib experiments in cell culture, drug was constituted in 100% dimethylsulfoxide (DMSO) at 1 mM stock, and was used at a final highest concentration of 0.1% DMSO.

### Mice

All animal experiments were approved by the University of Calgary Animal Care Committee and conducted following regulations established by the Canadian Council of Animal Care. Female C57BL/6 mice were purchased from Jackson Laboratories (Bar Harbour) for EAE experiments, and from Charles River (Montreal) for lysolecithin and cell culture experiments. Mice were housed in a specific pathogen-free facility.

***EAE***: For active EAE, 10–12 week old wildtype mice or CD45.1^+^ mice (Jackson Laboratories) were immunized with MOG35-55 peptide (50 μg/100 μL, Stanford University) emulsified in complete Freund's adjuvant (CFA) supplemented with 5 mg/mL heat-inactivated *Mycobacterium tuberculosis* H37Ra (Thermo Fisher Scientific); 50 μL emulsion was deposited on either side of the tail base. Pertussis toxin (PTX) (300 ng/200 μL; List Biological Laboratories) was injected IP on days 0 and 2 after MOG immunization. Vehicle or remibrutinib oral gavage was initiated 12 h after MOG immunization, and was repeated twice per day (BID) until the end of the study. Mice were scored for disability using a 15-point scale described previously [34]. At the harvest timepoint, mice received an i.p. overdose of ketamine/xylazine and were then transcardially perfused with 20 mL of cold PBS. The spinal cord was removed and the thoracic section was placed into 4% paraformaldehyde solution, then sucrose-protected and before cryosectioning longitudinally into 20 μm sections.

***Lysolecithin demyelination***: This was conducted as previously detailed [35]. In brief, a 34G needle attached to a 10 μL Hamilton syringe was inserted into the ventral spinal cord between the T3 and T4 vertebrae of young (10–12 weeks of age) or middle-aged (52 weeks) mice. 0.5 μL of 1% w/v lysolecithin (Sigma- L4129) was introduced at a rate of 0.25 μL/min and the needle was left in the spinal cord for 2 min to reduce backflow before it was removed. This protocol results in the prompt and near complete loss of cells (hours) in the lysolecithin-deposited area that then enabled repopulation of cells in the lesion [36]. As we were addressing regeneration of oligodendrocyte precursor cells (OPCs), which occurs from about 3 days after lysolecithin [35], daily oral gavage with vehicle or remibrutinib (60 mg/kg BID) was initiated at 48 h after injury. Mice were killed at day 10 for cryosections.

### Immunohistochemistry of tissue sections

Previously frozen spinal cord sections from EAE animals were permeabilized with 0.2% Triton X-100 and incubated overnight with a primary antibody against CD45 (BD Biosciences), then a fluorophore conjugated secondary antibody (Jackson ImmunoResearch Laboratories) and DAPI (Sigma) for 1 h, and mounted. Slides were imaged using an Olympus VS120 Slide Scanner with the 20x objective to capture the entire thoracic spinal cord. All slides were imaged with fixed exposure for each channel. Image analysis was performed in QuPath 6.0, where consistent intensity thresholds were set for each channel to accurately capture fluorescent signal. Total white matter area and lesions were annotated blind with manual tracing and measured for area in μm^2^. White matter lesions were identified using a combination of hypercellular DAPI + regions and CD45^+^ cell aggregates; dispersed CD45^+^ cells outside of hypercellular regions were not considered lesions. Lesion load was calculated as the ratio of the total lesion area to the white matter region of interest area for each sample.

For lysolecithin demyelination, coronal sections were stained with antibodies to PDGFRα (R&D Systems, AF1062), Olig2 (Millipore, ab9610) and CC1 (Millipore, APC) followed by their respective secondary antibodies from Jackson ImmunoResearch. Fluorescent images were analyzed using ImageJ software. The region of interest was identified by demyelination and elevated DAPI. The number of Olig2+ PDGFRα+ OPCs and Olig2+CC1+ oligodendrocytes were counted manually in a blinded manner within the lesion ROI.

### Myeloid cell studies in tissue culture

***Primary microglia and macrophages***: Human microglia were derived from fetal brain tissues obtained at legal abortions. The use of these specimens following parental consent was approved by The Conjoint Health Research Ethics Board at the University of Calgary. Human microglia were obtained using a previously described protocol [37]. Similarly, mouse microglia were obtained from the brain of neonatal pups [38]. Macrophages were prepared from the bone marrow of mice as described before [39]. All cell types were plated in 96-well plates for experiments. Cells were plated initially in 10% fetal bovine serum (FBS)-containing DMEM medium, and switched to 1% FBS medium 24 h before drug exposure. Remibrutinib or vehicle was then added for 5 h, followed without washout by 8 μg/mL immune complex {combination of ovalbumin (Invivogen) and anti-chicken egg ovalbumin (Sigma)} for 24 h. In optimization experiments, we determined that mouse microglia required 10 ng/ml interferon-γ (IFNγ) added to the immune complex for reliable elevation of TNF-α so the combination of immune complex and IFNγ was used to stimulate these cells. The medium was collected for TNF-α ELISA, and the plate was fixed with 4% PFA for subsequent staining with Iba1 for microglia, and phalloidin for macrophages. The stained cells were imaged on the ImageXpress Micro XLS High-Content Analysis System (Molecular Devices) using the 10x/0.5 NA air objective.

***iPSC-microglia:*** iPSCs (DYR0100) (ATCC) were differentiated into hematopoietic stem cells and further to microglia via a protocol optimized by Dorion et al. [40]. The induced microglia were used from day 28–35. Microglia were detached by Accutase and reseeded at 5000 cells/well in 100 μL of Medium 2.9 for experiments into a black-walled 96-well flat bottom plate coated with Corning® Matrigel® hESC-Qualified Matrix as per the manufacturer's recommendations. The following morning, cells were pre-treated for 5 h with remibrutinib followed by 100 ng/mL LPS and immune complex (IC 10 μg/mL) of ovalbumin and anti-ovalbumin antibody. The use of LPS on top of IC was because optimization experiments had suggested this combination for the iPSC-microglia to elevate TNF-α. While LPS is likely not present in the adult brain, toll-like receptor-4 activation (a downstream effect of LPS) is thought to occur in the brain in MS [41]. The conditioned media was then collected for ELISA, and the plate was fixed with 4% PFA.

### B cell isolation and polarization

The spleen and inguinal, axial, and brachial lymph nodes were dissociated, and B cells were then isolated using an EasySep Negative selection Mouse B cell Enrichment Kit (StemCell Technologies). Isolated B cells were then cultured in RPMI with l-glutamine supplemented with 10% FBS, 100 U/mL penicillin-streptomycin, 1x GlutaMAX, 1x MEM non-essential amine acids, and 50 μM β-mercaptoethanol at a concentration of 3 million B cells per mL at 37 °C 5% CO_2_. B cells were cultured in this media for 4 days, with media changes on days 2 and 3, with the following supplements to polarize B cells toward distinct cell types: BAFF protein (50 ng/ml), ODN1828 CpG nucleotides, IFNγ (50 ng/ml), goat anti-mouse IgM/IgG, and rat anti-mouse CD40 (5 μg/mL). Naïve B cells were generated by culturing isolated B cells with BAFF. T-bet^+^ memory B cells were generated by culturing isolated B cells with BAFF, murine IFNγ protein, ODN1828 CpG nucleotides, goat anti-mouse IgM/IgG and rat anti-mouse CD40.

For in vivo experiments using *in vitro* polarized B cells, the B cells were collected by centrifuging them at 500×*g* for 10 min at 4 °C after 4 days in culture. Cells were then washed with 40 mL of ice cold HBSS (Gibco), then centrifuged again. B cells were then suspended in ice cold HBSS again at a concentration of 50 million B cells per mL and 200 μL were injected intravenously into mice at peak disability and again 7 days later.

For *in vitro* experiments, B cells from individual mice were polarized for three days separately. Then on the third day the cells were split evenly into separate cultures. Either DMSO (vehicle) or remibrutinib (10 or 100 nM) were added to these cultures and 6 h later, the cultures were centrifuged and suspended in complete RPMI with BAFF protein and either vehicle or remibrutinib at the same concentration. Cells were then cultured for 24 h at 37 °C 5% CO_2_.

### Flow cytometry

On the day of tissue collection, mice were given a sublethal dose of ketamine/xylazine and perfused with 10 mL of ice cold PBS via injection into the left ventricle. Spleens cells were then collected. Spinal cords were extracted and pushed through 100 μm filters and suspended in a percoll (GE Healthcare Life Sciences) gradient (90%, 37%, and 30%) and centrifuged at 1800 rpm for 20 min at 4 °C. Immune cells were collected at the boundary between the 90% and 37% percoll interface. Cells were blocked with an anti-Fc-γ receptor (CD16/32 2.4G2, BD biosciences) and then stained with the following antibodies (clone) from BD biosciences: MHC2-BUV496 (M5/114.15.2), CD19-BUV563 (1D3), B220-BUV737 (RA3-6B2), CD11b-BUV805 (M1/70), TACI-BV421 (8F10), isotype-BV421 (R35-95), CD80-BV480 (16-10A1), and CD95-PE-Cy7 (Jo2); the following antibodies from Biolegend: CD4-BV510 (RM4-5), Ly6C-BV570 (HK1.4), Ly6G-PerCP (1A8), CD86-PE-Cy5 (GL-1), CD138-APC-Cy7 (281-2), and CD3 APC-Fire810 (17A2); and the following antibodies from Thermofisher: CD45.2-BUV395 (104), CD45.1-BV650 (A20), CD40-SB780 (IC10), CD38-FITC (90), and CD11c-PE-Cy5.5 (N418).

Cells were fixed for 20 min at 4 °C in 4% PFA, blocked with 4% rat serum for 20 min, and stained with the following antibodies from Biolegend: CD68-A700 (FA-11); and the following antibodies from BD biosciences: Tbet-PE (O4-46), and Blimp1-A647 (6D3) at 4 °C overnight. A defined number of counting beads (Invitrogen, C36995) was then added to the samples then washed 3 times with 1x permeabilization buffer. Cells were analyzed using a 5-laser Cytek Aurora. Spectral unmixing was done using Spectroflo (Cytek Biosciences) software then the data was analyzed using FlowJo (Treestar) software.

### ELISA and luminex analyses

Conditioned media was subjected to ELISA for mouse (Invitrogen) or human (R&D Systems) TNFα. For multiplex analyses, human samples were subjected to Eve Technologies' Human High Sensitivity T-Cell Expanded Panel 21-Plex Discovery Assay® Array while murine samples after 4 days in culture were evaluated using their mouse cytokine 32-plex panel.

### Statistical analysis

All statistical analyses were completed using GraphPad Prism 9. The particular statistical test used is described in the legend to the respective figures.

## Results

### Remibrutinib attenuates EAE disability and spinal cord inflammation

To examine if remibrutinib affects development of B cell-independent EAE, female C57BL/6 mice were immunized with MOG35-55. Mice were then oral gavaged with remibrutinib (60 mg/kg) or vehicle BID starting 12 h after immunization. The development of EAE disability was found to be similar in incidence between remibrutinib and vehicle-treated mice: 89 and 90%, respectively (p > 0.999, Fisher's exact test).

**Fig. 1:**
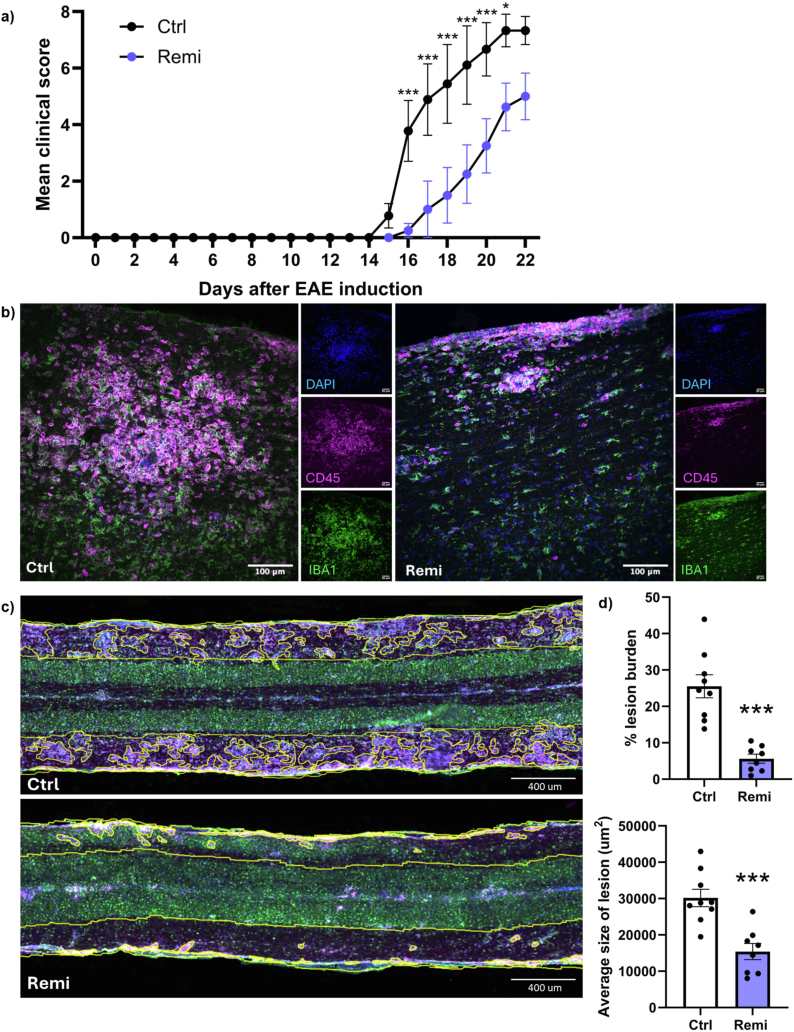
Remibrutinib attenuates the severity of EAE disability and spinal cord inflammatory lesion burden. Oral gavage of drug or vehicle was initiated 12 h after MOG immunization, and then twice daily until mice were killed for spinal cord analyses. (a) Daily disability scores analyzed by two-way (confounder of disability and time) ANOVA with Sidak's multiple comparisons. ∗p < 0.05, ∗∗∗p < 0.001. (b) Example images of lesion hypercellularity of DAPI + cells (blue) that were CD45^+^ (red), which were qualitatively larger in vehicle control (Ctrl) versus remibrutinib-treated EAE mice. (c) Slide scanner images of representative longitudinal spinal cord section from vehicle (top)- or remibrutinib (bottom)-treated EAE mice. The white matter on both sides of the central gray was analyzed with QuPath and the traced areas of lesions are displayed. Much more lesions are apparent in the vehicle-treated group. (d) Quantitation of inflammatory lesion burden in mice across the vehicle- and remibrutinib groups where each dot is data from an individual spinal cord. ∗∗∗p < 0.001 (two-way unpaired *t*-test). Values are mean ± SEM for panels a and d.

Vehicle-treated mice began to manifest EAE disability (loss of tail function) around day 15 after MOG immunization, and they progressed rapidly over the next few days with paresis and paralysis of the hind limbs (Fig. 1a). Mice treated with remibrutinib exhibited delayed EAE disability onset and reduced severity over the next few days compared to the vehicle-treated EAE mice.

All mice were killed at day 22 and thoracic sections of the spinal cord were assessed for lesional burden as defined by DAPI + hypercellularity and CD45^+^ immune density in the white matter. It was obvious that vehicle-treated mice had large clusters of CD45^+^ cells while those in remibrutinib mice were much smaller (Fig. 1b). To trace the area occupied by CD45+DAPI + cells, slide scanner images were evaluated as this provided a large area of the spinal cord for analyses. Qualitatively, the lesion burden was lower in remibrutinib compared to vehicle controls (Fig. 1c). The apparent lower immune lesion burden was corroborated by quantitation across animals for % lesion burden and the average size of lesion (Fig. 1d).

### Remibrutinib does not improve oligodendrogenesis following lysolecithin demyelination

**Fig. 2:**
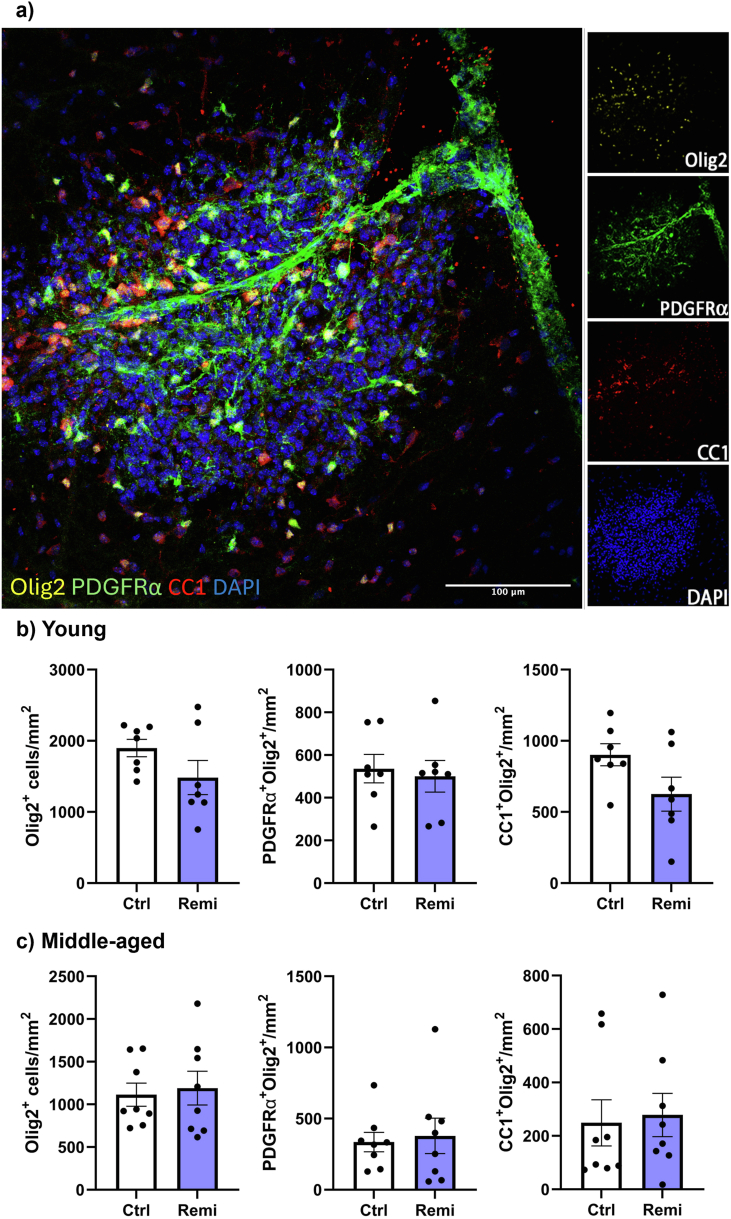
Remibrutinib does not promote oligodendrogenesis after lysolecithin demyelination. (a) Example of detection of oligodendrocyte lineage cells in the demyelinated area of the spinal cord in young mice showing merged (left) and individual panels (right). Olig2+ depicts oligodendroglial lineage cells, and doubly-positive Olig2+PDGFRα+ cells are OPCs while Olig2+CC1+ cells are oligodendrocytes. (b) Values do not differ significantly between the vehicle and remibrutinib group of young mice, and this was also the case for middle-aged mice (c). Each dot is data from an individual spinal cord (N of 7 for young mice and N of 8 for middle-aged mice in each group). Values are mean ± SEM.

Following the initial local depletion of cells by lysolecithin, new proliferating OPCs begin to repopulate the lesion and to mature into oligodendrocytes [35]. In young mice treated daily with 60 mg/kg remibrutinib from 48 h after injury and killed at day 10, there was no difference in the number of Olig2+ oligodendrocyte lineage cells, Olig2+ PDGFRα+ OPCs and Olig2+CC1+ oligodendrocytes between vehicle and drug-treated groups (Fig. 2a and b).

Since regenerative processes after lysolecithin demyelination is less robust in middle-aged compared to young mice [42,43], we repeated the experiment in middle-aged mice. Indeed, the number of Olig2+ cells in middle-aged lesions is half that of young mice (compare Fig. 2b and c). However, despite the lower baseline, remibrutinib did not elevate the number of Olig2+ cells, Olig2+ PDGFRα+ OPCs and Olig2+CC1+ oligodendrocytes in the middle-aged group (Fig. 2c).

### Remibrutinib reduces the level of cytokines in IC-activated primary microglia and macrophages, and iPS-induced microglia

**Fig. 3:**
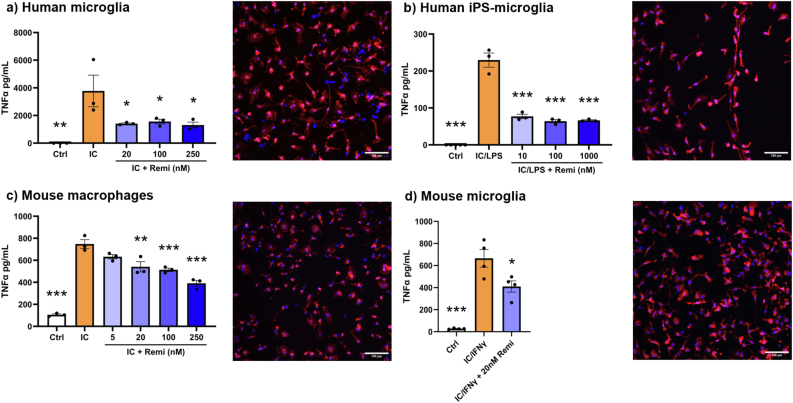
Immune complex (IC)-elicited elevation of TNF-α in myeloid cells, as an indicator of their activation, is attenuated by remibrutinib. This was the case for primary human microglia (a), human iPS-derived microglia (b), mouse bone marrow-derived macrophages (c) and mouse microglia (d). ∗p < 0.05, ∗∗p < 0.01, ∗∗∗p < 0.001 compared to IC (one-way ANOVA with Dunnett's multiple comparisons). An Iba1-stained image of each cell type is displayed, with the exception of macrophages that were stained with phalloidin (as mouse macrophages in culture stain poorly for Iba1), showing high similarity in morphology across all cultures. Values are mean ± SEM.

**Fig. 4:**
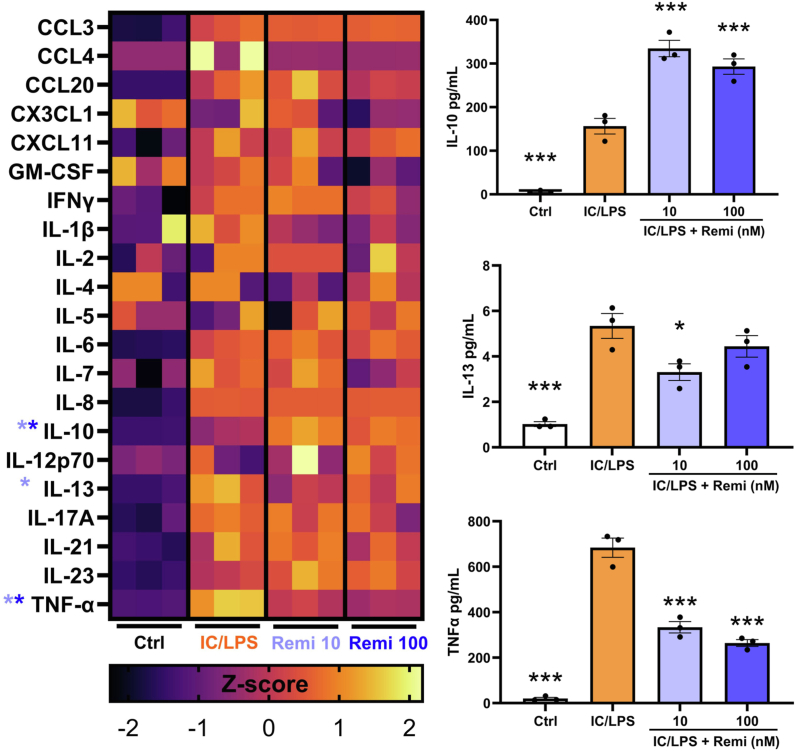
Multiplex cytokine analysis of conditioned media of iPS-derived microglia. Activation of cells with IC/LPS raised levels of most molecules (left), while the right panels display those significantly altered by remibrutinib. ∗p < 0.05, ∗∗∗p < 0.001 compared to IC/LPS (one-way ANOVA with Dunnett's multiple comparisons). Values are mean ± SEM.

Uniformly, across human and mouse primary microglia cultures and bone marrow-derived macrophages, remibrutinib reduced the amount of TNF-α elicited by IC-stimulation (Fig. 3). For human iPS-derived microglia, the secretion of TNF-α required the combination of IC and LPS (100 ng/mL) as either alone was insufficient; the IC/LPS-elicited rise of TNF-α was attenuated by remibrutinib (Fig. 3b). This was similar to mouse microglia which required co-stimulation with IFNγ (10 ng/mL) with IC to achieve a rise in TNF-α (data not shown). For all myeloid types, the remibrutinib attenuation was apparent from 10 or 20 nM, which is close to the IC50 concentration of this drug for BTK in isolated enzyme systems [26]. In multiplex Luminex assays, the conditioned media of IC-activated iPS-derived microglia also displayed lowering of TNF-α with remibrutinib. Additionally, IL-13 was lowered by drug while that of IL-10 was elevated (Fig. 4).

**Fig. 5:**
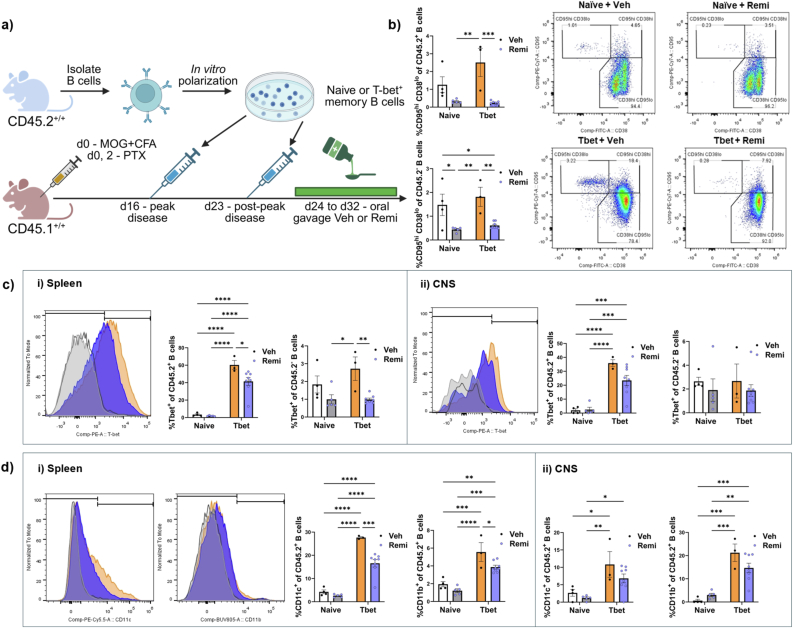
Remibrutinib affects T-bet^+^ memory B cells in vivo. (a) Schematic detailing the timing of intravenous B cell injections and oral gavage of remibrutinib or vehicle into EAE mice. (b) Quantification and representative flow plots of CD38 and CD95 expression on CD45.2^+^ transferred B cells (shown) and CD45.2^-^ endogenous cells to quantify the proportion of cells with a CD95^hi^ CD38^lo^ germinal center phenotype. (c) Expression of T-bet in CD45.2^+^ transferred B cells (shown) and CD45.2^-^ endogenous cells in the spleen (i) and CNS (ii). (d) Expression of CD11c and CD11b in CD45.2^+^ transferred B cells in the spleen (i) and CNS (ii). A two-way ANOVA was used to compare naïve B cell transfer and T-bet^+^ memory B cell transfer as well as whether treatment with remibrutinib or vehicle changed the measured variables; ∗p < 0.05, ∗∗p < 0.01, ∗∗∗p < 0.001, and ∗∗∗∗p < 0.0001. Values are mean ± SEM.

### Remibrutinib suppresses T-bet^+^ memory B cells in vivo

A population of B cells known as T-bet^+^ memory B cells has been hypothesized to be pathogenic in MS and may be affected by BTK inhibition [44]. To determine whether remibrutinib could affect naïve or T-bet^+^memory B cells in vivo, EAE was induced in CD45.1^+/+^ animals (Fig. 5a). At peak of disability and 7 days later, CD45.2^+/+^ B cells that were *in vitro* polarized into naïve or T-bet^+^ memory B cells were injected into CD45.1^+/+^ EAE recipients. After the second injection of T-bet^+^ memory B cells, mice were oral gavaged with either remibrutinib or vehicle for 9 days. Their spleens and spinal cords were then analyzed by flow cytometry. To confirm that the drug was active in the periphery of the body we assessed the proportion of transferred (CD45.2^+^) or endogenous (CD45.2^-^) B cells acquiring a germinal center phenotype (CD95^hi^ CD38^lo^) in the spleen as this process is BTK dependent [45]. Fig. 5b shows that remibrutinib suppressed the acquisition of a germinal center phenotype amongst both transferred naïve and T-bet^+^ memory B cells, and endogenous B cells, suggesting that the drug was active peripherally. Further analysis of B cells in the spleen identified that remibrutinib suppressed T-bet expression in both endogenous and transferred B cells, particularly in the transferred T-bet^+^ memory B cells (Fig. 5c). Consistent with drug suppressing phenotypes associated with T-bet^+^ memory B cells, remibrutinib also suppressed CD11b and CD11c expression on the transferred T-bet^+^ memory B cells (Fig. 5d). Similar trends were observed in the CNS but were not statistically significant.

### Remibrutinib inhibits T-bet^+^ memory B cell expansion and cytokine expression *in vitro*

**Fig. 6:**
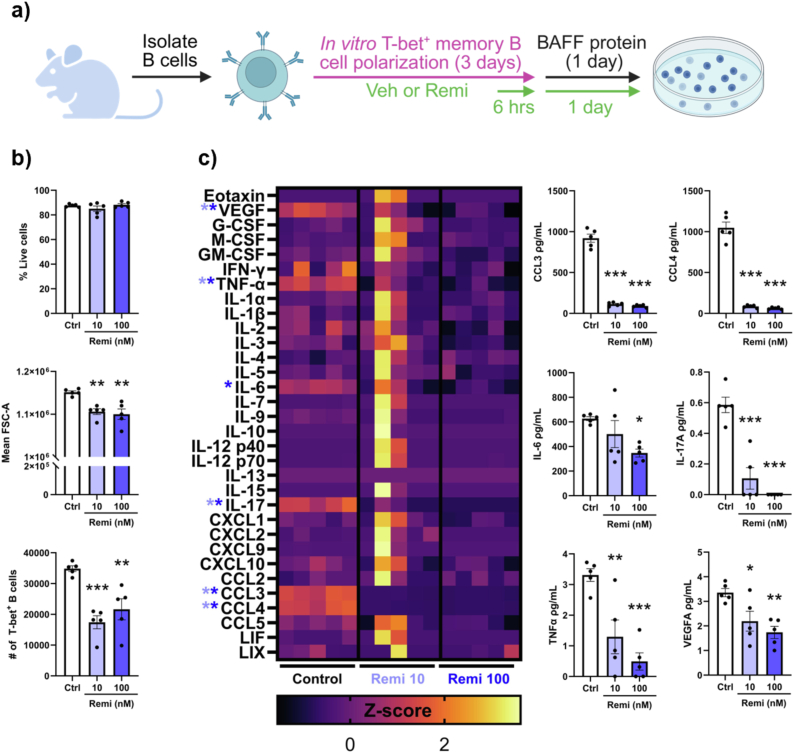
Remibrutinib affects T-bet^+^ memory B cells *in vitro*. (a) Schematic detailing the timing of remibrutinib or vehicle addition to *in vitro* T-bet^+^ memory B cell cultures (shown in green) and the removal of polarization reagents from the culture. (b) Quantification of flow cytometry results on the B cells at the end of the culture showing their viability, mean forward scatter area, and the absolute number of T-bet^+^ memory B cells. (c) Luminex analysis on the supernatants of the B cell cultures. The heatmap displays the Z-scored expression of the displayed cytokines and the graphs on the right show the absolute concentration of cytokines with statistically significant changes. Groups were compared using a one-way ANOVA with a Tukey's post-hoc test; ∗p < 0.05, ∗∗p < 0.01, ∗∗∗p < 0.001, and ∗∗∗∗p < 0.0001. Values are mean ± SEM.

To test precisely how remibrutinib was affecting T-bet^+^ memory B cells, we assessed how the drug would affect their *in vitro* polarization. To this end, naïve B cells were polarized into T-bet^+^ memory B cells for 3 days, then remibrutinib or vehicle was added to their culture media for 6 h (Fig. 6A). At the end of this incubation, B cells were washed and suspended in media without polarization factors, and only with BAFF survival factor and vehicle or remibrutinib, and cultured for 1 more day. Cells were then analyzed by flow cytometry, and their supernatants were analyzed by a 32-plex mouse cytokine panel.

The addition of remibrutinib to the cultures did not affect the viability of T-bet^+^ memory B cells; however, we did observe a reduction in the absolute number of cells recovered from remibrutinib treated cells (Fig. 6B). This reduction in cell numbers is likely to be driven by a blockage in cell proliferation as the mean forward scatter area, a measure of cell size that can be caused by cells blasting [46], of remibrutinib treated B cells was reduced. Further analysis of remibrutinib treated cells *in vitro* revealed no changes in the expression of CD38, CD95, CD11c, CD11b, CD80, and CD86 suggesting the expression of surface receptors was not affected by acute treatment (data not shown).

As T-bet^+^ memory B cells have been proposed to produce inflammatory cytokines [30,32] that could affect MS, we determined whether remibrutinib affected their capacity to make inflammatory cytokines. A luminex analysis of the culture supernatants from these B cell cultures revealed that remibrutinib reduced the production of VEGFA, TNF-α, IL-17A, IL-6, CCL3 and CCL4 (Fig. 6c). Thus, remibrutinib inhibits the biology of T-bet^+^ memory B cells.

## Discussion

Here, we found that a prospective medication for MS, remibrutinib, produces outcomes that are desirable in models of MS. Remibrutinib alleviates the extent of disability of EAE accompanied by reduced lesion pathology in the spinal cord, it lowers the pro-inflammatory activity of stimulated microglia and macrophages as informed by TNF-α level, and it inhibits the activity of T-bet^+^ memory B cells *in vitro* and in vivo. In iPS-derived microglia, measurement of several cytokines indicates a functional switch of cells to a regulatory anti-inflammatory state as manifested by elevated IL-10 and lowered TNF-α.

We did not find evidence for remibrutinib in promoting oligodendrogenesis following lysolecithin demyelination. Initial experiments utilized young mice where the repopulation of OPCs and oligodendrocytes after lysolecithin injury is spontaneously robust, and this could have masked a potential reparative effect for remibrutinib. Thus, we repeated the experiment in middle-aged mice with slower regenerative potential [43], but remibrutinib still did not enhance the lower rate of oligodendrogenesis occurring in these older animals. We note that a single BTK inhibitor, evobrutinib, has been reported to enhance myelin repair, but the models used were demyelinated cerebellar organotypic slice cultures or Xenopus MBP-GFP-NTR transgenic tadpoles [19,47]. More recently, however, evobrutinib was tested in the cuprizone model of demyelination where it enhanced the clearance of myelin debris by microglia, leading to accelerated remyelination [18]. More studies will have to be conducted to address whether the BTK inhibitors as a class, or particular ones, have pro-remyelinating responses which would be beneficial outcomes in MS.

It is now very clear that B lymphocytes are important drivers of disease activity in MS [6,48], especially since the B cell depleting anti-CD20 monoclonal antibodies profoundly reduce relapses and MRI activity in MS [49,50]. Recently, T-bet^+^ memory B cells, commonly called atypical memory B cells, have gained attention in MS as they concentrate in the CNS during disease [28], they can promote T cell activation through physical interactions [29], and they produce inflammatory cytokines [30]. Here we demonstrate that remibrutinib inhibits aspects of T-bet^+^ memory B cell biology in vivo and *in vitro*. We found that remibrutinib suppressed T-bet^+^ memory B cell expansion but did not affect their viability or phenotype *in vitro*. Consistent with our results, BTK inhibition in vivo has been found to reduce T-bet^+^ memory B cell proliferation [51] suggesting this phenomenon occurs *in vitro* and in vivo. In contrast to our results, others have found that adding the BTK inhibitor evobrutinib to human B cells being polarized into T-bet^+^ memory B cells leads to a reduction of CXCR3, CD11c, and T-bet expression [44] leading to fewer T-bet^+^ memory B cells being generated. An explanation for why these results differ from ours could be a difference in species (mouse vs human) but a more likely explanation would be differences in the timing of when the drug was added into the cultures. In our experiment, B cells were differentiated for 3 days prior to adding remibrutinib, ensuring that our cells had already differentiated prior to inhibiting their activity. In contrast, Rivers L. et al. [44] added evobrutinib to their cultures immediately and would have affected cellular differentiation. Thus, a likely difference between the experiments is that the latter is testing whether BTK inhibition would affect naïve B cells differentiating into T-bet^+^memory B cells whereas we tested whether BTK inhibition would affect atypical memory B cells once they had already formed. Indeed our *in vitro* data also demonstrated that BTK inhibition reduces the expression of the inflammatory cytokines CCL3, CCL4, TNFα, IL6, and IL-17a as well as VEGFA confirming that even after differentiating, BTK inhibition still affects the activity of T-bet^+^ memory B cells. This is consistent with the findings that phosphorylated BTK is constitutively higher in T-bet^+^ memory B cells relative to other B cell subsets due to chronic B cell receptor signaling occurring in these cells [51] suggesting that constitutive BCR signaling is important to their biology. It is also expected that the reduction in CCL3 and CCL4 expression by T-bet^+^ memory B cells may impact CD4^+^ T cell recruitment into the CNS as their supernatants have been shown to promote CD4^+^ T cell migration in *in vitro* experiments in part through these cytokines [32]. With well established pathogenic roles for TNFα, IL-17, and IL-6 in MS [52], these results suggest that BTK inhibition suppresses several mechanisms through which T-bet^+^ memory B cells could contribute to MS.

To determine whether our results could be extended in vivo we transferred our *in vitro* polarized T-bet^+^ memory B cells into EAE animals and treated the animals with remibrutinib for 8 days. Inhibition of BTK led to mild reductions in several markers associated with T-bet^+^ memory B cells including T-bet, CD11c and CD11b expression. Similar results have been seen when treating aged animals with BTK inhibitors for a period of 21 days where large reductions in T-bet, CD11b, and CD11c expression on B cells was seen [51]. Together these results suggest that chronic blockage of BTK signaling leads to a progressive loss in the T-bet^+^memory B cell signature. This may explain why in our *in vitro* experiment where atypical memory B cells are exposed to remibrutinib for a short period of time, that there was no loss in the expression of these markers as the assay was short. Overall, if T-bet^+^ memory B cells are pathogenic in MS, these results would suggest that remibrutinib is an effective therapeutic to suppress their activity.

Evidence do suggest that T-bet^+^ memory B cells are relevant to multiple stages of MS. The emergence of T-bet^+^ memory B cells with an inflammatory profile in the blood of individuals diagnosed with clinically isolated syndrome is predictive of conversion to MS, suggesting their involvement from early stages [30]. EBV associated with the development of MS also potently induces T-bet^+^ memory B cell differentiation [32]. Expression of T-bet in B cells has also been demonstrated to be required for an EBV-like virus to exacerbate EAE [31], suggesting these cells actively impact disease intensity. Few studies have looked at progressive MS; however, T-bet^+^ memory B cells have been found in the meninges of secondary progressive MS patients using single-nuclear RNA sequencing [29]. Consistent with the idea that these cells would contribute more as the disease progresses, T-bet^+^ memory B cells are known to increase with age [53] and age is the biggest predictor of MS progression [54]. Mechanistically, there are a number of ways that T-bet^+^ memory B cells may be contributing to MS. Several studies have now associated an inflammatory cytokine profile with T-bet^+^ memory B cells and thus these cells could be modifying the inflammatory environment through the production of inflammatory factors [30,32]. T-bet^+^ memory B cells also readily differentiate into plasma cells [55], which seems to be the case in MS [29] and could explain the accumulation of plasma cells in the CNS of MS patients as the disease progresses [56]. Lastly, interactions between T-bet^+^ memory B cells and CD4^+^ T cells in the periphery of the body have the capacity to drive the proliferation of autoreactive T cells and their accumulation into the CNS, a process that occurs in relapsing-remitting and progressive MS [29,57]. Taken together, T-bet^+^ memory B cells are likely associated with all stages of MS and could be contributing through several mechanisms.

There are limitations to our study. In the EAE and lysolecithin models, outcomes of remibrutinib were assessed in acute disease, while long term treatment should have been conducted where other outcomes including repair might have become evident. Another limitation is that our study does not address an area of considerable importance in MS neurodegeneration, that is, whether BTK inhibition affects relapse-associated worsening and progression independent of relapse activity. However, such MS-specific phenomena should best in addressed in clinical trials as animal models of MS are not sophisticated enough to display these features.

In summary, while the results of remibrutinib in Phase 3 trials of MS are awaited, it is reassuring that the drug favorably affects the inflammatory outcomes of models that are pertinent to MS.

## Authors contributions

RWJ, AR, KH, MTM, GR, MM, XZ and CL provided data for this manuscript. MNN and FG supplied inducible-pluripotent stem cells and the microglia derived from these cells. BG advised on the handling of remibrutinib and other perspectives of this drug. MX and VWY provided oversight and the whole program was supervised by VWY. All authors read and edited the manuscript. VWY finalized the article.

## Declaration of competing interest

BC is an employee of Novartis BioMedical Research, Basel, Switzerland. VWY has received speaker honoraria from Biogen, EMD Serono, Novartis, Roche and Sanofi-Genzyme. All other authors declare no conflicts.
